# Optical near-field mapping of plasmonic nanostructures prepared by nanosphere lithography

**DOI:** 10.3762/bjnano.9.144

**Published:** 2018-05-23

**Authors:** Gitanjali Kolhatkar, Alexandre Merlen, Jiawei Zhang, Chahinez Dab, Gregory Q Wallace, François Lagugné-Labarthet, Andreas Ruediger

**Affiliations:** 1 Institut National de la Recherche Scientifique - Énergie, Matériaux, Télécommunications, 1650 Boulevard Lionel-Boulet, J3X 1S2, Varennes, Québec, Canada; 2 IM2NP, UMR CNRS 7334, Aix Marseille Université et Université de Toulon, Site de l’Université de Toulon, 83957 La Garde Cedex, France; 3 Institut Fresnel UMR 7249, Aix-Marseille Université, CNRS, École Centrale de Marseille, 13013 Marseille, France; 4 Western University (The University of Western Ontario), Chemistry Department and Centre for Materials and Biomaterials, 1151 Richmond Street, London, ON, N6A5B7, Canada

**Keywords:** apertureless scanning near-field optical microscopy, diffuse signal, nanosphere lithography, photomultiplier tube, plasmonic nanostructures

## Abstract

We introduce a simple, fast, efficient and non-destructive method to study the optical near-field properties of plasmonic nanotriangles prepared by nanosphere lithography. Using a rectangular Fourier filter on the blurred signal together with filtering of the lower spatial frequencies to remove the far-field contribution, the pure near-field contributions of the optical images were extracted. We performed measurements using two excitation wavelengths (532.1 nm and 632.8 nm) and two different polarizations. After the processing of the optical images, the distribution of hot spots can be correlated with the topography of the structures, as indicated by the presence of brighter spots at the apexes of the nanostructures. This technique is validated by comparison of the results to numerical simulations, where agreement is obtained, thereby confirming the near-field nature of the images. Our approach does not require any advanced equipment and we suggest that it could be applied to any type of sample, while keeping the measurement times reasonably short.

## Introduction

SNOM (scanning near-field optical microscopy) is an imaging technique based on an optical near-field probe for high spatial resolution [1–2]. A version of this technique is the apertureless scanning near-field optical microscopy (aSNOM), also known as scattering scanning near-field optical microscopy (s-SNOM). aSNOM has demonstrated tremendous potential for the study of nanostructures, as it combines the extreme sensitivity of surface enhanced optical analysis with the high spatial resolution of scanning probe microscopy [3–5]. This method has been applied to various fields of research such as plasmonic analysis [6–7], Raman spectroscopy (tip-enhanced Raman spectroscopy, TERS) [8–9], or infrared analysis [10]. In this microscopy technique, a laser is focused onto a nano-antenna consisting of a noble metal tip, whose role is to enhance and to confine the near-field and to transform it into a measurable far-field signal. An electromagnetic near-field amplification is generated in the process. The main challenge is to fabricate highly efficient tips to ensure a detectable near-field contribution that will exceed the ubiquitous background far-field signal in the scattered light. In ideal conditions, fast scanning on the surface ensures a high sensitivity, and minimizes the drift. To achieve this objective, most nano-antennas used in aSNOM are based on plasmonics nanostructures. The highly enhanced and confined electromagnetic field created at the apex of the tip through the resonant surface plasmon excitation results in a very high spatial resolution, far below the diffraction limit.

Nevertheless, aSNOM still encounters some challenges. The most important one is to guarantee a high near-field contribution in the scattered signal, as the near/far-field magnitude ratio is usually critical and strongly affected by several experimental parameters. In addition, artefacts can lead to misinterpretation of the results. They can have several origins, such as topography effects [11], tip defects [12], or interferometric effects [11]. Consequently, the resulting high far-field background signal tends to overcast the near-field contribution of the signal. To address these drawbacks, several methods to enhance the near-field contribution have been developed [13]. Most of them are based on a combination of experimental development and post treatmemt analysis of the recorded pictures. One approach consists in removing the background signal using anharmonic detection techniques [14–15]. An alternative consists in amplifying the signal using homodyne [16], heterodyne [17] or pseudoheterodyne [18] interferometry. However, these methods are challenging to put in place and require complex analytical devices. In certain cases, the interpretation of the pictures is far from being straightforward.

In this work, we introduce a simple method to image the near-field contribution in aSNOM pictures for periodic plasmonic structures. We focus on the elastically backscattered signal measured through a photomultiplier tube (PMT) with an aSNOM setup in conjunction with gold tips that were used as local probes. To remove the periodic background far-field contribution, we use the Fourier transformation of the diffuse signal and filtering. This method is applied to plasmonic gold nanotriangles [19] inscribed on a glass substrate, that were prepared by nanosphere lithography [20–22]. Those nanostructures, that most commonly consist of arrays of metallic (gold or silver) nanotriangles deposited on a glass or Si substrate, are of high interest to study plasmonics, and more specifically localised surface plasmon resonance (LSPR) [23–24]. Indeed, their geometry and their metallic nature result in the spatial confinement of the electric field at their apexes and referred as hot spots [25–26]. After data processing, the far- and near-field contributions are separated and the hot spots are revealed unambiguously, even though convoluted with transient hot-spots due to the plasmonic nature of the scanning probe tip. The images recorded using this method are compared to numerical simulations by finite elements. A good agreement is found, attesting to the validity of our method of analysis and making it an alternative technique to study the hot spots in plasmonic nanostructures.

## Results and Discussion

**Figure 1:**
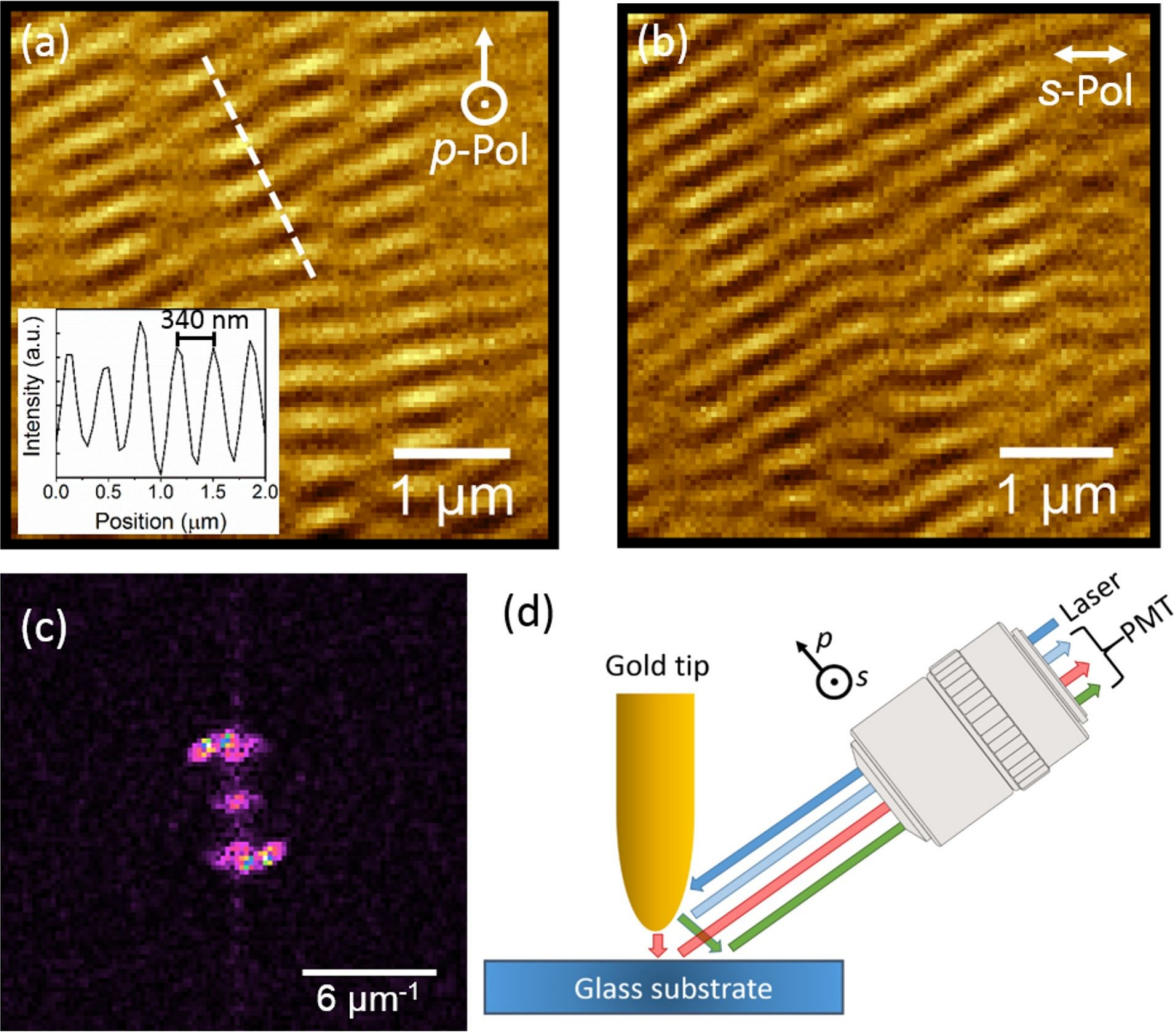
Diffraction patterns measured on glass for (a) the *p*-polarization, the inset illustrates a line profile of the intensity oscillations along the dashed white line in (a), and (b) the *s*-polarization of the laser. (c) 2D FFT of the *p*-polarized optical image, and (d) schematic of the confocal detection.

**Figure 2:**
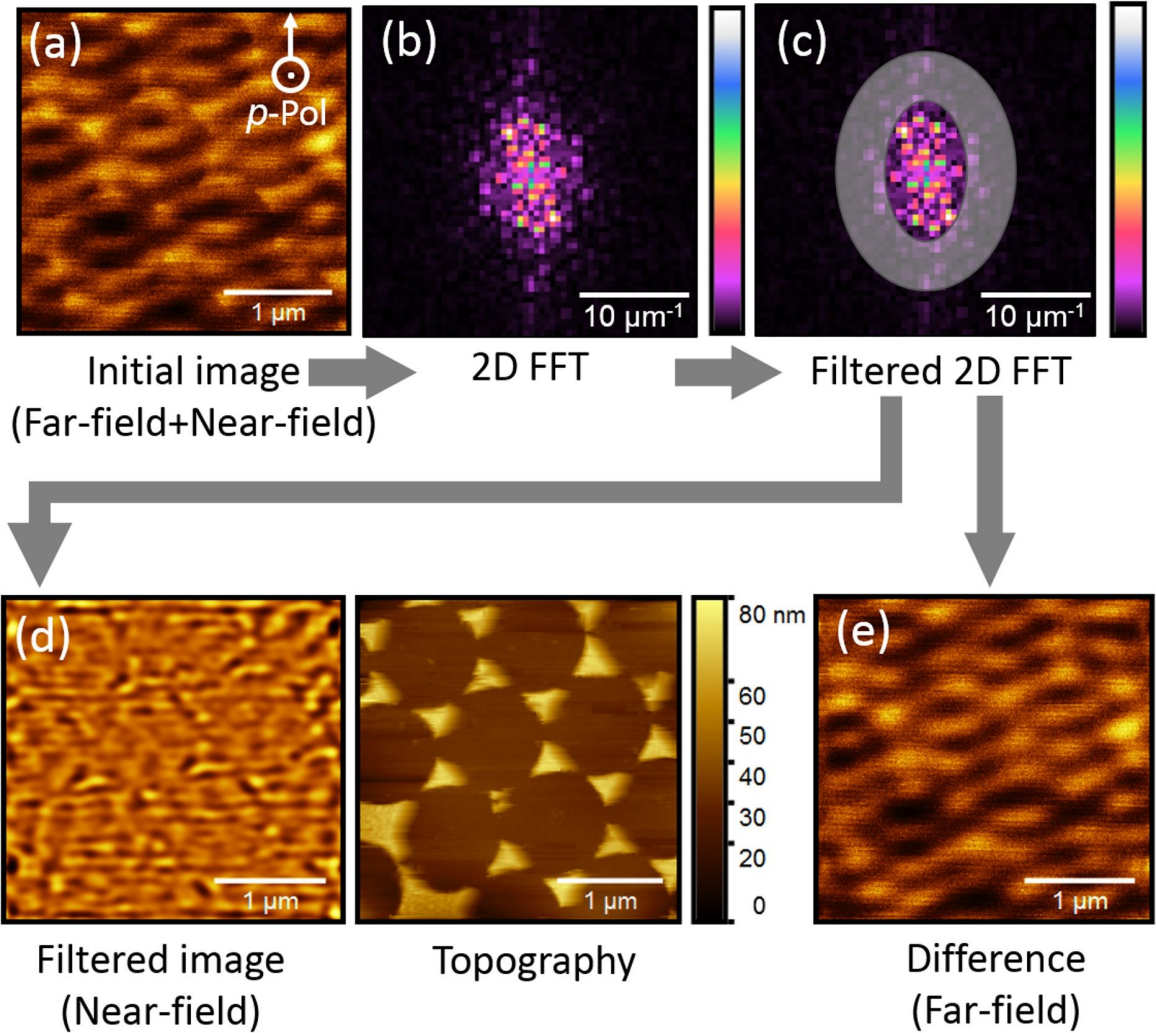
Schematic of the analysis performed on the optical images, showing (a) the initial optical image, (b) the 2D FFT of the optical image, (c) the 2D FFT image where the frequencies to be retained are highlighted (grey disc), (d) the filtered optical image obtained after the FFT analysis and the corresponding topography and (e) the difference between the initial optical image and the filtered image.

As mentioned previously, the near-field information can be challenging to extract from the optical images due to a high background caused by the far-field signal, the magnitude of which often buries the near-field contribution. To confirm the presence of the far-field component, aSNOM measurements were first performed on a flat glass substrate. With such a sample, the optical signal should not show any significant variation over its surface. However, Figure 1a and Figure 1b demonstrate an inhomogeneous optical response consisting of optical oscillations. For an excitation of 632.8 nm, a periodicity of ≈340 nm is measured, which translates into a periodicity of ≈3 µm^−1^ in the Fourier (2D reciprocal) space. The anisotropy of this pattern is also reflected in the 2D fast Fourier transform (FFT) of the optical image, presented in Figure 1c. In addition, those patterns vary with the polarization, and with the laser focus. Thus, these oscillation patterns can be attributed to an interference phenomenon between the light scattered by the tip, the tip–sample interaction and the sample itself, as illustrated in Figure 1d. All these contributions combine to form a complex interferometric pattern recorded by the PMT. But this pattern does not contain any near-field contribution coming from the sample itself. As can be seen in Figure 2a, the complex interferometric background is also present with the gold nanotriangles. This feature prevents the direct observation of their optical near-field contribution.

To extract this near-field signal from the images we used an approach based on an FFT filtering process. Indeed, the far-field contribution seen in Figure 1 is characterized by low spatial frequencies, whereas the highly confined near-field contribution should be associated to higher spatial frequencies. Using the FFT of the recorded image enables us to discriminate far-field against near-field. The analysis is schematically described in Figure 2. This approach is generally applicable as optical interference phenomena necessarily occur at low spatial frequencies due to the diffraction limit.

First, the 2D FFT of the image is calculated to display the different frequencies composing the initial image (Figure 2b). In Figure 2c, a rectangular bandpass filter is used to retain spatial frequencies between 3 and 8 µm^−1^. The frequencies below ≈3 µm^−1^ are due to the interferometric background of the glass substrate, while those above ≈8 µm^−1^ are attributed to the noise of the system. For an undistorted image, a hexagonal filter pattern would be most appropriate. The fact that the micromanufacturing process did not cause any noticeable contaminations however allows for the use of a more convenient filter option with angular isotropy. A filtered image with noticeably reduced oscillations is obtained (Figure 2d). This filtered image better displays the near-field contribution of the optical signal. It can be used to directly map the hot spots and to correlate their spatial positions to the topography of the investigated structures. Figure 2d shows the area of the nanoscale triangles where the field is confined. The apexes as well as the bases of the triangles can be identified as areas where the incident electromagnetic field is confined as localized surface plasmon modes. They match the location of the original arrays of gold triangles as seen in the topography. The difference between the initial and the filtered images displays the far-field oscillations and is very similar to the non-treated optical image that contains both the far- and near-field contributions (Figure 2e). This implies that the pure near-field contribution is very weak and post-data treatment is imperative to retrieve the near-field information.

**Figure 3:**
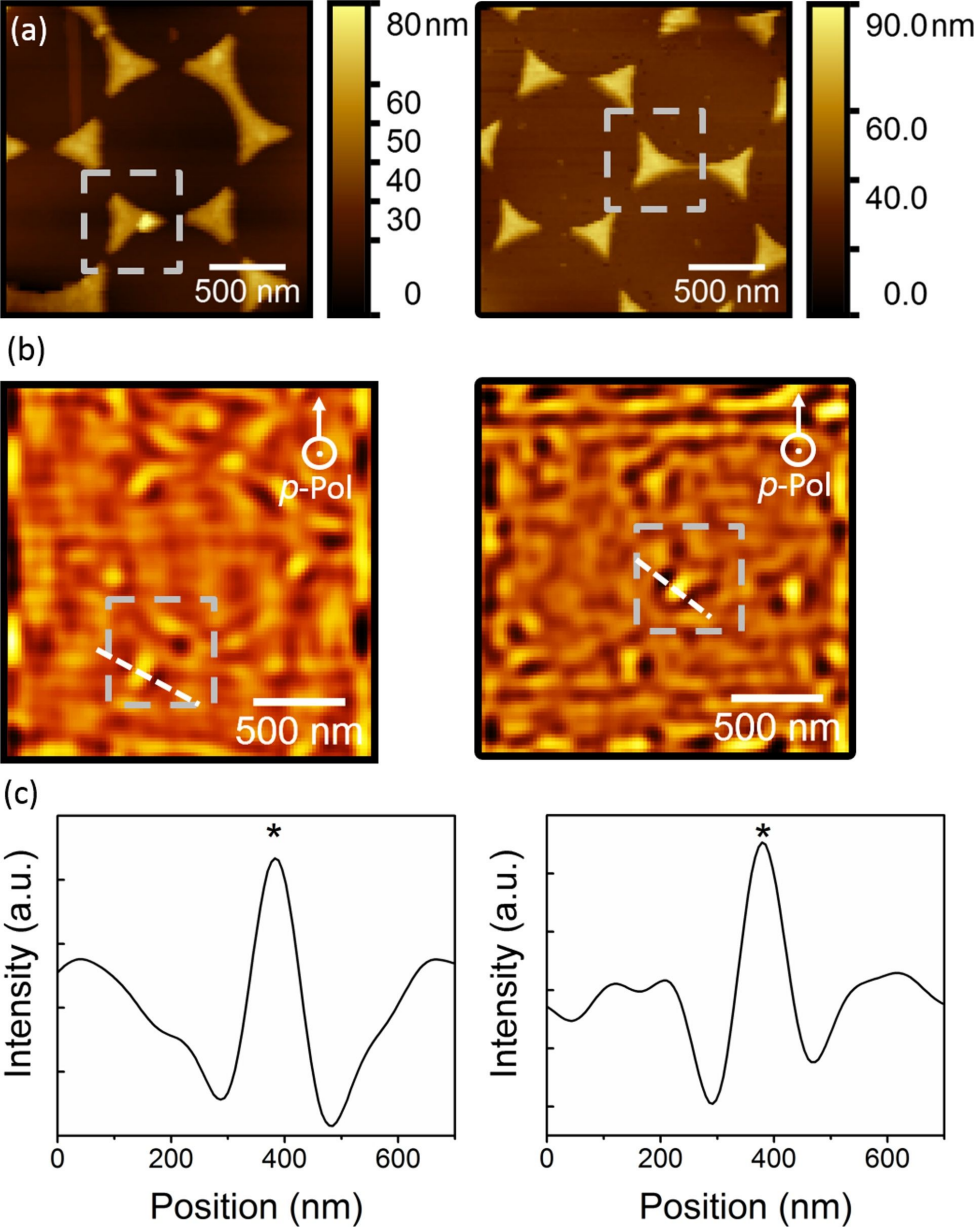
(a) Topography maps acquired on a nanotriangle array, (b) PMT images obtained after filtering, and (c) corresponding profiles across the apex of a nanotriangle (*), using red (632.8 nm) and green (532.1 nm) *p*-polarized laser excitations, respectively.

We used this protocol to study the optical near-field properties of gold nanotriangle arrays with two different laser wavelengths of 632.8 nm and 532.1 nm, using the same pixel size (20 nm) and a *p*-polarization, as presented in Figure 3. The change in wavelength does not significantly affect the optical images. In both cases, a good agreement between the topography (Figure 3a) and the structures appearing after the filtering (Figure 3b) can be found, attesting to the quality of the filtering. Some parts around the nanotriangles appears brighter. It has been shown in previous studies that such features could be associated to topographic artifacts. Yet, instead of systematically appearing at the edges of the plasmonic nanostructures, the brighter regions are located at the apexes of the nanotriangles while the rest of the edges remain darker (Figure 3b). Figure 3c displays profiles acquired across the apex of a nanotriangles using both excitation wavelengths. In both cases, the intensity of the signal increases at the plasmonic structure’s apex. A spatial resolution of ≈100 nm was estimated for both pictures using the full-width at half maximum of the peaks. This value is defined by the high pass filter cut-off frequency (≈8 µm^−1^ in reciprocal space, ≈125 nm in real space). Note that our scheme is not primarily sensitive to the intrinsic, static nanotriangle resonance (in the 500–850 nm range). The plasmonic nature of our scanning probe gold tips introduces transient hot spots as the tip scans over the surface. The fact that a strong enhancement is observed indicates that the resonance of the capacitive gap mode between the tip and the surface is close to the laser excitation wavelength.

**Figure 4:**
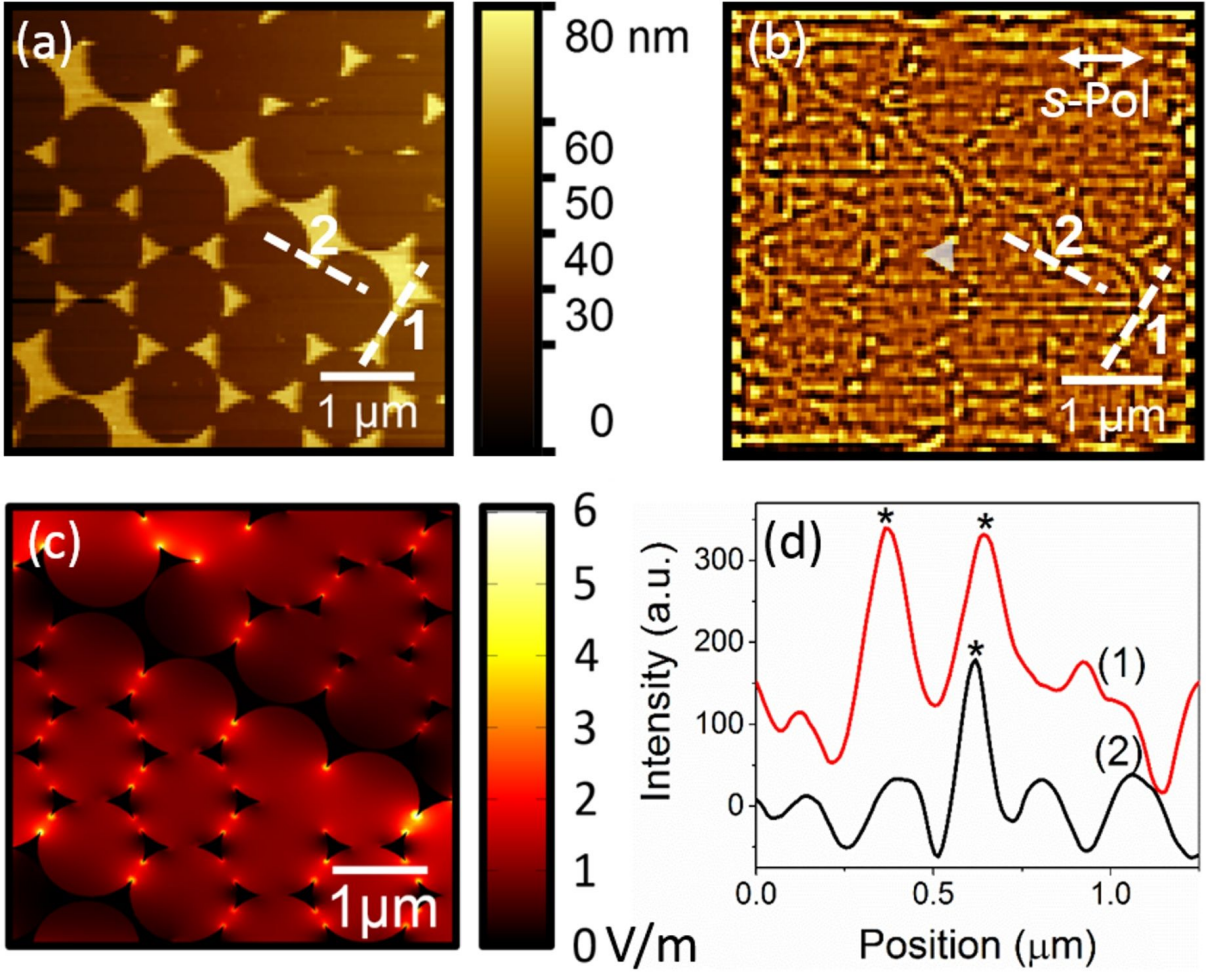
(a) Topography and (b) PMT image after filtering obtained with the 632.8 nm *s*-polarized laser excitation, where an example of a nanotriangle is highlighted (c) corresponding numerical simulations, and (d) profiles of the experimentally measured optical image taken across hot spots, where “*” indicates the position of a hot spot (the intensity shift between the two profiles was added for clarity).

**Figure 5:**
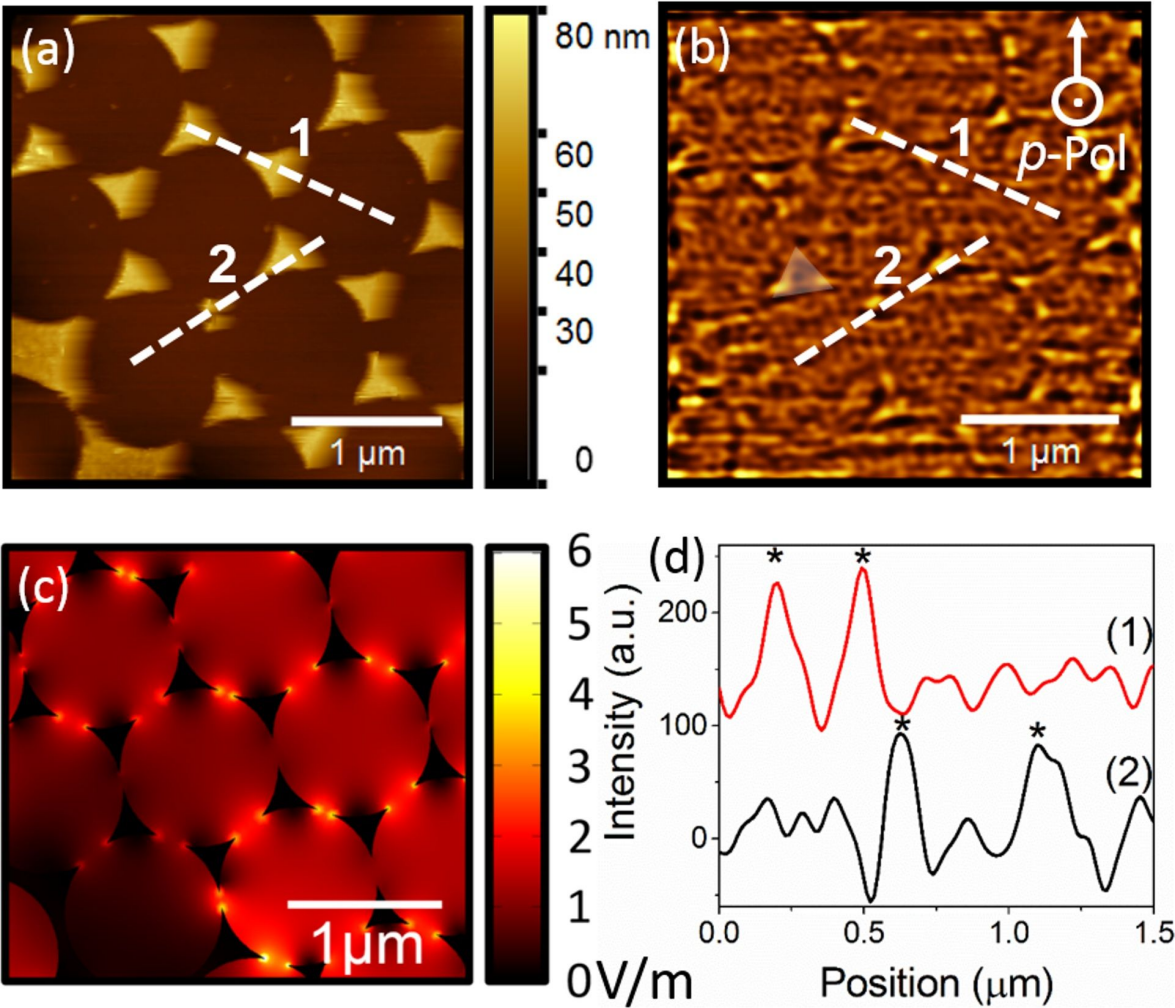
(a) Topography and (b) PMT image after filtering obtained with the 532.1 nm *p*-polarized laser excitation, where an example of nanotriangle is highlighted (c) corresponding simulations, and (d) profiles of the experimentally measured optical image taken across hot spots, where “*” indicates the position of a hot spot (the intensity shift between the two profiles was added for clarity).

The measurements were then conducted on larger areas using different polarizations (Figure 4 and Figure 5). Consistent with Figure 3, a clear correlation is obtained between the topography (Figure 4a and Figure 5a) and the filtered PMT images (Figure 4b and Figure 5b), where the outline of the circles formed by the nanospheres appears darker, while the apexes of the nanotriangles, corresponding to the hot spots, appear brighter. The features of the processed images can be extracted with a high spatial resolution confirming that we have a near-field image, as a diffraction limited image could not reach such a high resolution. It is worthwhile mentioning that the cut-off of the filtering process ultimately determines the spatial resolution: a compromise needs to be found to remove the high frequency noise without removing the near-field signal as noticed above.

Surprisingly, no polarization effect was observed. Our configuration provides a confined and strongly enhanced electromagnetic field at the tip apex. It has been shown in TERS measurements that depolarization mechanisms can be induced by the tip [27] while the exact physical origin remains unclear. We suggest that this feature accounts for the lack of polarization dependence in our experiments.

The same nanotriangle patterns and setup configuration were simulated for both wavelengths, as presented in Figure 4c and Figure 5c. The hot spots observed on the simulated images are located at the same position as on the experimental images, after the filtering. Due to the orientation of the nanotriangles with respect to the laser beam polarization, hot spots appear at all three apexes of the nanotriangles, consistent with the experimentally acquired near-field images. This supports the statement that the images obtained after the FFT treatment are of near-field nature.

Furthermore, on the filtered images, the hot spots do not only appear at the apex of the nanotriangles, but on other structures such as the gold surfaces observed in Figure 4a. All the nanostructures found on the surface contribute to the electromagnetic enhancement, which is visible in our images. With our technique, we can see precisely where the hot spots are located. Two profiles taken across hot spots, as described by the white dashed lines on the topography (Figure 4a and Figure 5a) and filtered images (Figure 4b and Figure 5b), are presented for both wavelengths in Figure 4d and Figure 5d. The peaks indicate the position of the hot spots. The average intensity on the surface without hot spot is close to 0. On the hot spots, the intensity increases to ≈200 at 632.8 nm and ≈100 at 532.1 nm, revealing that in our measurements, the amplifications are very low. They are much lower than in the simulations, which is ascribed to different reasons: for one, the experimental radii of the apexes might be larger than in the numerical model, or the crystallinity of the gold composing the nanotriangles might be less than ideal thus causing dissipation. Also, the simulation assumes perfectly crystalline gold. In this study, the samples here were prepared by evaporation and have no defined crystallinity, unlike colloidal solutions composed of monocrystalline metallic particles which generally provide higher enhancements by several order of magnitude [21]. However, the enhancement of the optical near-field is clearly visible, demonstrating the potential of our technique.

Other methods were previously used to characterize the hot spots on plasmonic nanotriangles. They can be compared with our approach. One technique consists in irradiating the samples with short laser pulses to achieve plasmon-mediated ablation [23]. The resulting local melting reveals the hot spots, but at the cost of the sample destruction. Other methods use surface enhanced Raman spectroscopy (SERS) [28–29].

In these cases, a Raman active molecule is deposited on the surface of the sample. Its Raman signal is only visible on the hot spots due to higher and localized enhancement. Nevertheless, this technique is diffraction limited to a few hundred of nanometers as it is based on confocal microscopy measurements. Galarreta et al. [26] coated such triangular structures with an azopolymer thin film sensitive to laser irradiation and induced surface deformation at the hot spots upon laser illumination. The localization of the hot spots is then indirectly revealed in the subsequent AFM scanning of the surface [26]. Similarly, Murazawa et al. [30] spin-coated a pattern of gold nanorods with a photoresist before irradiating the sample with a femtosecond laser, thereby inducing polymerization at the hot spots. In another approach, based on confocal fluorescence microscopy, a dye solution is deposited on the samples, and the higher fluorescence magnitude originating from the hot spots is measured [19]. However, these last four techniques lead to sample contamination due to the use of a molecular marker. Other techniques based on electron–sample interactions can be utilized to non-destructively map the distribution of the localized plasmon modes. In photoemission electron microscopy (PEEM), the sample is irradiated with a femtosecond pulse laser, generating electron–hole pairs that are confined at the apex of the nanotriangles. The electron emission is imaged, and local variations in this emission results in an image contrast. The photoemission is strongly enhanced by the LSPR excitation at the hot spots, resulting in a higher intensity on the map [31]. The same principle is used with scanning transmission electron microscopy (STEM) and electron energy loss spectroscopy (EELS) [32–34]. This technique based on inelastic scattering measures the energy losses of the electrons. At the hot spots, the plasmon excitation results in a decrease in the energy of the incident electron that can be imaged by EELS. However, these electronic techniques require conductive samples. Also, the surface preparation is challenging, as the sample has to be thinned for the measurements. An alternative approach consists in using aSNOM with lock-in or heterodyne detection to enhance the near-field signal [35–36]. While these techniques were able to successfully perform optical imaging, the data analysis remains highly complex. Compared to these methods, our approach offers several advantages: It is a non-destructive technique that does not require complex experimental devices, it provides fast measurements with high resolution, and can be applied to virtually any kind of sample. No additional treatment is required, and no chemical solution is employed, avoiding sample contamination.

## Conclusion

In summary, we measured the optical near-field properties of nanotriangles using backscattering signal measured by a PMT. Through a simple analysis method based on FFT image filtering, we were able to isolate the near-field contribution of the signal. After the treatment, the hot spots appear brighter and their position can be identified within 100 nm precision. The analysis was performed on measurements taken with two different laser wavelengths, and no significant difference in both spatial resolution and field distribution was observed. High spatial resolutions of down to approximately 100 nm were obtained, this final resolution being a compromise between the efficiency of the filtering and the attended resolution. The obtained images were in good agreement with numerical simulations reproducing the experimental configuration. Our method offers several advantages such as efficiency, simplicity and rapidity. It underlines that optical near-field measurements can be easily performed without heavy experimental devices or complicated posttreatment analysis.

## Methods

### Sample preparation

The samples were fabricated by nanosphere lithography. A solution of 1 µm diameter polystyrene microsphere mixed with ethanol was deposited on a dry, clean microscope glass coverslip and distributed homogeneously. After the sample dried, 3 nm of Ti followed by 30 nm of Au were deposited using electron beam evaporation. The sample was then sonicated in ethanol for 1 min to remove the polystyrene particles. A pattern of gold plasmonic nanotriangles (side length ≈350 nm, thickness ≈33 nm) with a P_6mm_ symmetry was obtained [19]. The optical absorption of the sample was measured: the nanotriangles array exhibit a broad resonance between 500 and 800 nm.

### Experimental setup

The near-field studies were performed using an AIST-NT OmegaScope 1000 TERS system equipped with a Nanofinder 30 Raman spectrometer and a thermoelectrically cooled CCD detector. A TEM00 cw He–Ne laser (632.8 nm) and a cw solid-state Cobolt 04-01 series laser (532.1 nm) were used as the excitation sources. A 0.7 N.A. Mitutoyo MPlan Apo 100× objective placed under a 65° inclination was used to focus the laser onto a gold tip. A schematic of the experimental setup can be found elsewhere [37]. The elastically backscattered signal detected by a PMT was studied here.

To obtain a tip with a radius down to 10 nm and low surface roughness, the gold tips were fabricated by electrochemical etching. A 100 µm thick gold wire (Goodfellow, Purity 99.99+ Annealed) was immersed to a depth of ≈1 mm in a 37% hydrochloric acid (HCl) solution (anode) in the center of a gold ring (cathode). A pulsed voltage (30 µs, 3 kHz frequency) of 7.5 V peak to peak and a +0.5 V offset was applied to the tip. The etched tip was then glued onto an Abracon Corporation, AB38T-32.768 kHz tuning fork operated in shear force configuration.

### Image treatment

To perform the FFT analysis of the optical images we used the open source software Gwyddion [38]. Any AFM software offering these processing options could be used to achieve the same results. Advanced filter functionality including non-rectangular frequency filters might require a dedicated data analysis software.

### Simulations

The numerical simulations were done with COMSOL Multiphysics using the finite element method. To reproduce the experimental conditions, a 632.8 nm *s*-polarized, or 532.1 nm *p*-polarized, laser beam at 65° from the sample surface was focalized on a nanotriangle array. Triangular free and fine meshes, with a minimum element size equal to 1.2 × 10^−10^ m and a curvature factor of 0.2, were used to discretize the geometry under analysis, and in every single mesh, Maxwell’s equations were solved. The triangular meshes are surrounded by a matched layer with a distribution of a fixed number (8 in this case) of mapped elements to strongly absorb the outgoing waves created in the computational region instead of reflecting them back inside.
